# Supplementary material for: Association Between the Jiangnan Diet and Mild Cognitive Impairment Among the Elderly
Source: Nutrients. 2025 Oct 10;17(20):3189. doi: 10.3390/nu17203189 (PMC12566854; doi:10.3390/nu17203189)
Supplement: Supplementary file 1 [file nutrients-17-03189-s001.zip › nutrients-3883907-supplementary.pdf]

Supplementary Table 1 Sensitivity analysis of association between Jiangnan diet and MCI

Variables	Q1	Q2	Q3	Q4
No adjusted	1.00	0.68(0.47~0.99)	0.42(0.29~0.63)	0.45(0.31~0.67)
Age adjusted	1.00	0.69(0.47~1.00)	0.44(0.29~0.65)	0.46(0.31~0.68)
Multivariate adjusted*	1.00	0.53(0.36~0.80)	0.32(0.21~0.50)	0.21(0.12~0.36)

*Adjusted for age, frequency of social activities, depression, hypertension, alcohol consumption and energy intake.
